# Implementing An External Student Placement Strategy Into an Undergraduate Chiropractic Curriculum in the United Kingdom: An Education Descriptive Report

**DOI:** 10.1016/j.echu.2023.01.001

**Published:** 2023-05-31

**Authors:** Paul Chesterton, Faye Deane, Daniel Moore

**Affiliations:** School of Health and Life Sciences, Teesside University, Middlesbrough, United Kingdom

**Keywords:** Chiropractic, Education, Health Education, Students

## Abstract

**Objective:**

The aim of this descriptive report is to describe the development and implementation of a placement strategy into an entry-level chiropractic course within the United Kingdom.

**Methods:**

Placements are educational experiences during which students can observe or apply theory in real practice situations. For this study, the placement strategy was developed for the chiropractic program at Teesside University through an initial working group that generated its aims, objectives, and philosophy. Evaluation surveys were completed for each module containing placement hours. The median and interquartile range (IQR) were calculated for combined responses using a Likert scale (1 = strongly agree; 5 = strongly disagree). Students were allowed to provide comments.

**Results:**

A total of 42 students participated. Placement hours were divided across all taught years (Academic Year 1: 11%; Year 2: 11%; Year 3: 26%; Year 4: 52%). Data were evaluated 2 years post-launch, with 40 students reporting to be satisfied overall with Year 1 (median 1, IQR 1-2) and Year 2 (1, IQR 1-2) placement modules. Participants perceived that placement experiences were applicable to the workplace and their future careers across modules in both Year 1 (1, IQR 1-2) and Year 2 (1, IQR 1-1.5) and that continuous feedback improved their clinical learning (Year 1 [1, IQR 1-2]; Year 2 [1, IQR 1-2]).

**Conclusion:**

This report describes the strategy and student evaluation findings over its 2-year inception, exploring the principles of interprofessional learning, reflective practice, and authentic assessment. The strategy was implemented successfully following placement acquisition and auditing processes. Student feedback reported overall satisfaction with the strategy, which was associated with graduate-ready skills.

## Introduction

Health care placements are educational experiences during which students can apply theory in real practice situations under the supervision of qualified professionals.^1^ It is pertinent that graduating students are exposed to an adequate volume and variety of patients during entry-level education in order to meet statutory body requirements and expose them to a wide range of clinical situations they may experience after graduation.^2^ Chiropractic students have traditionally received clinical education within an institution-based clinic, with only some programs offering hospital-based clinical placements.^3^, ^4^, ^5^ A limited format of clinical education has been criticized for lack of breadth and depth.^6^ As a solution, clinical immersion placements (CIPs) provide practical hands-on experiences aimed at developing communication and interpersonal skills within the community.^7^ Clinical immersion placements have the potential to provide chiropractic students with a diverse patient caseload underpinned by a caring environment.^8^ It is hypothesized that exposure to community clinics may provide a superior educational experience, thus preparing students for the challenges faced in current practice.^9^

External clinical education and placements are an extension of university-led teaching, which may allow students to experience the reality of the health care industry, facilitating the transition of theory into practice while immersing themselves in the profession's values.^10^ In addition to standard institutional teaching, placements develop students’ clinical competence while strengthening skill sets and self-confidence.^10^^,^^11^ Benefits extend to the faculty, with clinical educators solidifying their knowledge base, stimulating their own learning opportunities while challenging them to re-evaluate and improve their practice.^12^ Experiential learning in this context through educator clinical experience links theory, practical experience, and professional development to provide a complete educational experience.^13^ The challenge for entry-level courses is providing an authentic and enriched learning experience that prepares students for clinical practice.^14^

Traditionally, in the United Kingdom, allied health professions (eg, physical therapists, occupational therapists, speech and language therapists) include external placements alongside theoretical teaching throughout the life span of the course. Chiropractic programs in the United Kingdom tend to rely on university clinics, with limited variation provided externally by private and hospital-based settings across all 4 years of the course. Therefore, the chiropractic program at Teesside University aimed to develop a novel and innovative United Kingdom strategy of clinical experience delivery, with experience of both private and public sector health care settings, providing an environment for interprofessional observation. The placement strategy was underpinned by the principles of interprofessional learning (IPL), reflective practice, and authentic assessment. The concept of a formal placement strategy mapped horizontally (across year modules) and vertically (throughout course levels) to learning outcomes was unique within chiropractic education in the United Kingdom. This formal placement strategy introduced students early in their education to a sustained clinical caseload, which was entrenched throughout the entirety of the curriculum.^6^

The aim of this descriptive educational report is to outline how this placement strategy was created and implemented, detailing its variations from CIPs and isolated final-year internships. The report evaluates the success of the strategy, through student evaluation, over its 2-year inception and how the principles of IPL, reflective practice, and authentic assessment have been embedded.

## Methods

Following implementation, a questionnaire of undergraduate student's experiences in the chiropractic program at Teesside University between September 2020 and May 2022 was conducted. All data were collected through internal audit/module evaluation processes in accordance with the standard University policy service evaluation.

### Ethics

No additional data were captured, and therefore formal ethical approval was exempted by the School of Health and Life Sciences at Teesside University. This report is written in accordance with the education descriptive report outline.^15^

### Initial Working Group

A working group of stakeholders was appointed to support the development of the course structure. This included 3 local practicing chiropractors, a representative of the Society for Promoting Chiropractic Education, and a faculty Head of Department. This group informally investigated the viability of students completing clinical hours through placements within clinics external to the University. All potential placements were considered within (1) the principles of IPL, (2) reflective practice at its core, and (3) the provision of an authentic experience/assessment. The success of IPL hinges on commitment, faculty engagement, and the design, support, and delivery of the program.^16^ Therefore, in addition to the IPL delivered on campus, the placement strategy sought to expose students to a range of health care professions in different clinical environments. Reflective practice, a dynamic and changeable process, is a cornerstone of health care education, intrinsically linked to the quality of student learning,^17^ and was integral to the strategy. The stakeholder group also identified the need for an authentic assessment experience with pedagogic flexibility and creativity, adding value to the student experience.^18^ Initial placement strategies were presented to local clinics and patients, gaining informal feedback presented at hosted meetings by the working group, refining the overall approach.

### Independent Scoping Exercise

The University recruited an independent expert (who was a practicing chiropractor and General Chiropractic Council Education Committee member) to conduct a virtual placement scoping exercise of clinics within 120 miles of the main campus. This exercise collected pertinent data in respect of private chiropractic clinics, such as location, registration, and philosophical beliefs, in addition to potential public health care setting such as regional National Health Service foundation trusts.

Clinics were assessed based on predetermined criteria. Clinics were considered to be an appropriate placement opportunity for entry-level education if their websites provided current, evidence-based information about chiropractic practice and were given a “green” rating. Clinics whose websites included non-evidence-based information were categorized as a “red” rating. If enough information was not available to make a judgment, the clinic was not assigned a rating and was left to be further investigated. Overall, 126 locations were identified in the scoping exercise; 14 clinics were identified as green and 16 as red.

### Initial Placement Acquisition Process

Starting in March 2020, the chiropractic course leader engaged clinic owners in discussion for the purpose of identifying potential placement locations. Clinics and placement providers closest to the university's main campus with green ratings were contacted. The process from initial contact to being a placement provider was performed in 3 steps.

#### Initial Discussion

The chiropractic course leader contacted clinic owners and explained the placement process, audit process, training, and commitment levels required. External chiropractic clinic owners and associated health placement providers asked to consider the value of collaboration and if they wanted to progress to placement audit.

#### Placement Audit

A clinic visit was arranged, and a University Audit Document was completed (see audit form in supplementary material). Any missing policies/procedures or information were identified, and advice was provided about how to achieve compliance. All participating third-party, public liability insurance policies (those related to non-personal injury claims not associated with treatment) were examined. The University's student medical malpractice insurance document, which covered claims for patients’ personal injury related to associated assessment and treatment, was shared. Potential chiropractors’ registrations were checked against the United Kingdom General Chiropractic Council's website, ensuring no ongoing disciplinary action was active. Previous disciplinary records were not accessed.

#### Practice Educator Training

A placement coordinator ensured all practice educators received initial assessor training prior to hosting students. Training was supported through an online support site, which each educator was encouraged to work through prior to accommodating students. The training served as a useful reference tool providing the necessary documents required by placement educators, including a placement educator checklist, handbook, Practice Assessment Document (PAD), Practice Placements & Practice Environment Profiles and continuing professional development dates.

The strategy included bi-annual clinical liaison meetings, which provided an opportunity for the chiropractic placement lead, course leader, and practice educators to evaluate collectively and analyze progress. This combined review provided an authentic opportunity to discuss ongoing development.

**Fig. 1:**
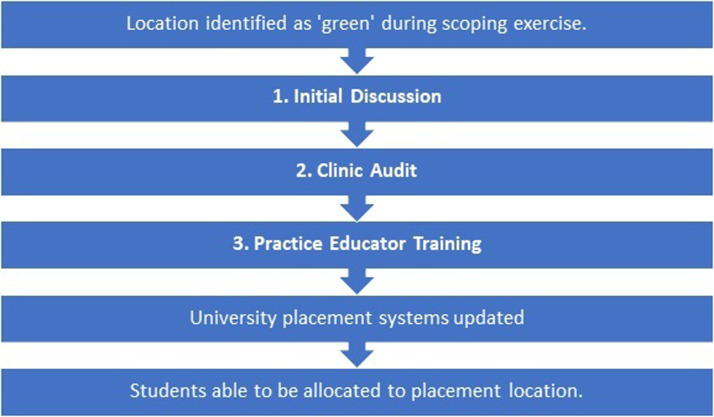
Placement acquisition flowchart overview.

Following completion of the 3 steps, clinics were successfully added to the placement providers’ placement software (PEP Net Placement), internally held at the University. At this point, the clinic became eligible to receive students on placement. A standardized monetary remuneration scheme with external providers was agreed upon. Figure 1 provides an overview of the process.

### Implemented Placement Strategy

The placement development strategy was integrated within the entry-level Master of Science (Hons) chiropractic degree. The curriculum, described as a sequence of learning experiences, was developed and approved following service-user and community partnership collaboration. The placement strategy was aligned with learning outcomes, syllabus, assessment, and learning and teaching pedagogy.^19^

Table 1 presents the total number of placement hours at each academic year and the percentage of placement hours across the lifespan of the degree. There is currently no minimum number of placement hours stipulated within the Education Standards of the regulatory body, The General Chiropractic Council.^20^ A competency model opposed to a “minimum” hours-based model was therefore implemented. The breakdown of the number of hours was based on clinic capacity, the academic calendar, and conflicting educational priorities within other taught modules.

**Table 1:** Placement Hours Across the Course

Academic Year	Placement Frequency	Total Hours	Percentage of Placement Hours
1	10 d (4 d in chiropractic setting, 6 d in NHS setting)	74	11
2	10 d (chiropractic setting)	74	11
3	26 d (chiropractic setting)	182	26
4	52 d (chiropractic setting)	364	52
Total	98 d	694	100

*NHS*, National Health Service.

#### Academic Year 1

Placements were embedded across the academic modules, which introduced students to core clinical and professional competencies. These included subjective assessment, chiropractic manual skills, and understanding the role of the chiropractor and other health care professionals. Summative assessment was categorized as pass or fail and was subject to achievement of learning outcomes detailed on the PAD (see assessment document in supplementary material). These include but were not limited to respectful verbal and non-verbal communication, punctuality, appearance, attitude, professionalism and respect, and consideration of a patient's privacy, dignity, and cultural differences.

Before entering a practice placement experience, students completed mandatory in-house training, including health and safety evaluation, risk assessment, safeguarding, conflict resolution, and information governance. Students spent 4 days in an observatory capacity with an external chiropractic placement provider. They spent an additional 6 days within a National Health Service (NHS) environment, which is a government-funded medical and health care service in the United Kingdom. Chiropractors did not operate within the NHS setting but were able to gain experiential and reflective experiences in Academic Year 1. From Year 2 onward, only chiropractic placements were utilized to ensure students were exposed to a relevant clinical caseload. While on NHS placement days, students were embedded as part of a therapeutic care support team on hospital wards, including rehabilitation, orthopedic and acute frailty.

#### Academic Year 2

Students spent 10 days in a chiropractic setting under supervision of a practice educator, where they were given a subjective assessment process to facilitate the planning of a comprehensive chiropractic objective assessment. A summative assessment (pass/fail) was awarded based on feedback gained from placement educators via the PAD (see assessment document in supplementary material). This method developed an authentic assessment strategy, which aimed to facilitate learning throughout the placement experience, rather than award students a final arbitrary grade.

#### Academic Years 3 and 4

The learning outcomes across these academic years refined clinical reasoning skills and enhanced evidence-based decisions regarding diagnosis, differential diagnosis, and plan of management. Evaluation of performance under supervision from a registered chiropractor of both subjective and objective assessment skills and clinical management plans was evaluated over 26 days in Year 3 and 52 days in Year 4. This was completed via an on-campus University outpatient chiropractic clinic.

Summative assessment was categorized as pass/fail based on the learning outcomes, in addition to a student's ability to effectively analyze and interpret the chiropractic assessment findings, develop appropriate patient-centered management plans, justify selected treatments, assess outcome measures, and evaluate treatment outcomes. This was achieved via a competency-based Practice Assessment Tool, which was completed over the placement duration and was focused on achieving key competencies and demonstrating these to chiropractors acting as clinical tutors.

### Student Data Analysis

Following implementation, students across both academic year groups (1 and 2) were asked to complete a module evaluation survey, which was a standardized anonymous online survey used to deliver course and module evaluation. Students were asked to agree or disagree (5 = strongly agree; 1 = strongly disagree) on statements related to their learning experience. The survey was managed by a central administration community that did not teach the Master of Science (Hons) chiropractic course. The survey was not developed specifically for the placement modules and had been used previously. Questions with Likert scales were reported as numeric variables, with the median and interquartile range (IQR) calculated for combined responses across potential answers. Students were also allowed to provide comments.

## Results

Forty-two students were enrolled in the Master of Science (Hons) chiropractic course and participated in the placement strategy during the initial 2 academic years (n = 30 Year 1; n = 12 Year 2).

### Student and Placement Feedback

The results of the end of the module survey are reported in Table 2.

**Table 2:** Student Module Evaluation Data

Academic Year	Semester	Number of Enrolled Students, n	Number of Students Who Completed Survey, n (%)	Staff Have Made the Subject Interesting, Median (IQR 25-75)	This Module Challenged Me to Do My Best Work, Median (IQR 25-75)	Feedback Has Helped Me Develop and Improve My Learning, Median (IQR 25-75)	Module Provides Me With Experiences Applicable to the Workplace, Median (IQR 25-75)	I Am Satisfied With the Quality of This Module, Median (IQR 25-75)
1	1	30	27 (90)	1 (1-2)	2 (1-2)	1 (1-2)	1 (1-2)	2 (1-2)
	2	30	21 (70)	1 (1-2)	2 (1-2)	1 (1-2)	1 (1-2)	2 (1-2)
2	1	12	9 (75)	1 (1-2)	1 (1-2)	1 (1-2)	1 (1-1)	1 (1-2)
	2	12	7 (58)	1 (1-1)	1 (1-1.5)	1 (1-1)	1 (1-1.5)	1 (1-1)

Likert scale: 1 = strongly agree; 2 = agree; 3 = neutral; 4 = disagree; 5 = strongly disagree.

Opportunities to obtain feedback from qualified clinicians during the placement strategy were informal and formal. Informal comments provided during the clinical encounter delivered timely and specific feedback. During Semester 1, feedback centered on advice improving non-verbal body language and self-efficacy. Semester 2 feedback adopted a feedforward approach acknowledging the improvement of performance in a progressive and constructive manner. The following is example feedback from the same educator across 2 semesters for 1 student:

“Be mindful of non-verbal communication, you were sometimes closed off by crossing your arms and legs. Think about more open posture—it will make you feel more confident too!” (Year 2 Student Feedback, Semester 1, Placement Visit 1)
“X is aware of her own anxiety and has taken positive steps to plan and manage it—well done! Asked engaged questions. An improvement in non-verbal communication this week. Very good friendly and relaxed attitude with patients.” (Year 2 Student Feedback, Semester 2, Placement Visit 5)
Students were able to add qualitative comments at the end of module evaluations. While these comments were limited, they provided feedback on the placement strategy.
“The Pass/Fail element removes pressure in trying to attain a high mark. It allowed me to enjoy the learning experience.” (Year 1 student)
“Really interesting module and provides a lot of opportunities for feedback and practical development.” (Year 2 student)
“Continual reflection has developed my own understanding of subjective assessment.” (Year 2 student)
“Enjoyable and engaging module, particularly the practical time where we can gain insights from multiple clinicians which are transferrable to the workplace.” (Year 2 student)

## Discussion

We implemented a novel United Kingdom chiropractic student placement strategy underpinned by the principles of IPL, reflective practice, and authentic assessment. Key findings from this descriptive educational report suggest that students agreed that the placement experience challenged them to produce their best work while instilling experiences, which were integral to the profession. Additionally, students felt that the placement strategy ensured the learning experiences were interesting and reported overall satisfaction with the quality of the modules.

### Student Feedback

The placement strategy was developed to nurture students’ skills in relation to their core knowledge while supporting the transition to patient management, building confidence, and reducing the fear and anxiety graduates often experience. The ethos and philosophy of the strategy encouraged the scaffolding of student learning within authentic learning environments under the supervision of highly skilled and experienced chiropractors. Students’ feedback across the first 2 implementation years suggests that they were satisfied with the quality of the placement modules and perceived these to be intrinsically linked to the workplace.

The external placement strategy enabled students to receive in-context formative and summative feedback in real time through a developmental lens. Previously feedback was provided post-summative assessment in a proxy-generated clinical environment (University viva/practical examination), whereas the current strategy provided constructive clinical feedback allowing students to incorporate a reflective strategy to promote meaningful learning.^21^ Incorporating these placement experiences early in a student's learning journey reinforced theoretical components in a real-world context. We felt that it was important to create a longitudinal opportunity for reflection and personal clinical development starting in the first year of the course.

### Interprofessional Learning

Central to our strategy was IPL, where educators or learners from more than 1 health care–related discipline jointly created and fostered a collaborative learning environment.^22^^,^^23^ Students with IPL training value interprofessional-focused patient health care.^24^ IPL has been reported to have a range of benefits in relation to integrated patient care, professional identity, communication, and teamworking across a range of disciplines.^25^, ^26^, ^27^ Knieper et al^28^ have identified the need for chiropractic students to be exposed to IPL opportunities to enhance and enrich the learning experience. Chiropractic students have identified the importance of structured collaborative education in their own health care development.^28^

The strategy described in this narrative aimed to integrate an interprofessional method of learning throughout the student journey. Students may benefit by developing “soft” or interpersonal skills. These baseline skills are integral for students to prepare themselves for graduate work.^29^ Ultimately, the goal of the strategy was aligned with this vision by training chiropractic students to work as a part of the health care team rather than as independent clinicians and to generate better health outcomes for the wider community.^30^

### Reflective Practice

Autonomous chiropractic professionals may be a product of their clinical education and experiential learning within entry-level teaching.^3^ The variety of clinical cases that chiropractic students are exposed to may be limited and, therefore, may not be reflective of true practice.^31^^,^^32^ Reflecting upon clinical encounters promotes critical thinking, thus narrowing the gap between theory and practice and stimulating personal development.^33^^,^^34^

The placement strategy allowed students to immerse themselves within the clinical environment, from the start of their course, with strong reflective scaffolding embedded throughout. Students’ qualitative responses suggested they were able to develop key clinical skills through a reflective process. The benefit of continual tutor interaction and peer-to-peer discussions centered around their experience in practice, which we believe may have produced an engaging metacognitive reflective process. Throughout Academic Years 2 and 3, reflective writing was embedded within students’ assessment portfolios with a focus on self-reflection from feedback gleaned both intrinsically and extrinsically from the placement educators. During Year 4, the reflective process encouraged students to identify specific learning needs measured against core clinical competencies during the clinical consultation. The goal was to encourage autonomous, self-directed learning, synonymous with that of Continuing Professional Development frameworks provided by the General Chiropractic Council in the United Kingdom.

### Assessment

Placing assessment at the heart of practice through a pass/fail approach was adopted to provide the most authentic assessment method of student assessment. Several students suggested a pass/fail approach removed the emphasis of striving for a high-graded mark at the end of their placement experience, allowing them to focus on the learning opportunities. Student assessment within an environment familiar to their graduate context demanded significant pedagogic flexibility in the design. Practice educators evaluated interpersonal skills in a live environment where students were inherently consumed with patient-centered care approaches. This provided a genuine and relatable assessment method in which students showcased their professional attitudes and behaviors in a variety of environments (eg, private vs public health centers, primary vs secondary care)

Feedback received by students was an “intrinsic” and meaningful directive for students to reflect upon.^35^ To ensure a quality experience for students, chiropractic educators familiarized themselves with the core curriculum and the level of expected student competencies.

The authors of this manuscript argue that assessment allowed evolving feedback and was a helpful learning strategy for students. The results from the module evaluations in this report suggest that students at least agreed that the feedback given in real time had facilitated their professional development. We based this assumption that feedback was an important catalyst for improvement but was only beneficial to learning if students were active participants acknowledging, reflecting, and acting upon it.^36^

### Challenges to Implementation

Several challenges were experienced during implementation. There was a limited number of clinic providers who were willing to accept student placements. The coordination of students completing mandatory training, necessary pre-placement paperwork, and organizing travel arrangements was time-consuming. The training of placement educators, ensuring students were exposed to high-quality care and chiropractic practices within an evidence-based framework, added workload burdens.

### Limitations and Future Research

The placement strategy described was taken from the design and implementation at only 1 United Kingdom Higher Education Institution. Subsequently, the number of students who participated in the placement strategy was relatively low (n = 42), and therefore our findings may not be generalizable. The robustness of the strategy over time requires further evaluation through interviews and focus groups. Evaluation of multiple cohorts as they complete the various stages of the strategy, listening to the student's voice through both quantitative and qualitative research designs, is needed to develop a clearer picture of its effectiveness. Future research should formally evaluate the perspectives of all stakeholders, including faculty, external educators, service users, and students. This UK study may not reflect the nuances of international institutions that operate in a different educational context.^37^ Understanding the reasons why qualified chiropractors want to engage in the education process may also create future collaboration opportunities. The longer-term benefits to students are likely to be multifaceted, and while initial feedback is positive, future research involving all stakeholders is required to understand the longevity of the strategy and its impact on student outcomes and success.

## Conclusion

This report describes the development and implementation of a formal external placement strategy embedded within an entry-level course in the United Kingdom. The strategy was underpinned by the principles of IPL, reflective practice, and authentic assessment. Students in this sample were satisfied with the placement and felt it challenged them to undertake their best work. Qualitative comments suggested students linked the skills learned through the strategy to those required on graduation.

## Appendix Supplementary materials

Image, application 1
