# Supplementary material for: Implementing An External Student Placement Strategy Into an Undergraduate Chiropractic Curriculum in the United Kingdom: An Education Descriptive Report
Source: J Chiropr Humanit. 2023 May 31;30:1–8. doi: 10.1016/j.echu.2023.01.001 (PMC10247877; doi:10.1016/j.echu.2023.01.001)
Supplement: Supplementary file 1 [file mmc1.pdf]

Supplemental File

Implementing an external student placement strategy into an undergraduate chiropractic curriculum in the United Kingdom: An education descriptive report

Paul Chesterton

Faye Deane

Dan Moore

Journal of Chiropractic Humanities

Supplementary Material

University Full Placement Audit Standards.

Are the following policies/procedures available in the practice placement area and are staff and students made aware of them?		
**Required**	**No**	**Yes**
Health and safety		
Incident/accident reporting		
Ionising radiation regulations		
Risk assessment including students under the age of 18		
Confidentiality and consent		
Equal opportunities		
Moving and handling		
Medicines management		
Infection prevention and control		
COSHH		
Adult / Child Protection		
Lone worker policy		
Fire / emergency procedures		
Bullying and harassment		
Raising and escalating concerns/whistle blowing		
Code of conduct for Nurses and Midwives (current NMC code – refer to NMC website)		
Guidance on Professional conduct for Nursing and Midwifery Students (current NMC guidance – refer to NMC website)		

Practice Assessment Document

Module: CHI1002-N- Foundation Skills for Chiropractic Practice 1

STUDENT: ....

This form can be used for more than one professional, however, if extra space is needed a separate form may be submitted.

Day	Date	Location	Hours AM (e.g.4)	Educator Name	Hours PM (e.g.3.5)	Signature
1						
2						
3						
4						
5						

		Achieved	Not-achieved	
		(please tick Appropriate Box)		
	**PROFESSIONAL CONDUCT**			**Comments / Recommendations for Development**
	Demonstrates adherence/ integration to unit policies & routines & confidentiality			
	Demonstrates appropriate appearance, attitude, and professional behavior			
	Is able to manage time effectively including punctuality			
	Demonstrates responsibility & initiative within the practice setting			
	Is able to reflect and recognise their own personal limitations and the scope of their practice			
	Demonstrates an inquisitive approach within the practice setting			
	**COMMUNICATION & INTERPERSONAL SKILLS**			
	Demonstrates appropriate and respectful communication & liaison with relevant staff members			
	Demonstrates respect and consideration of patients' privacy, dignity and cultural differences			
	Demonstrate appropriate and professional verbal communication skills			
	Demonstrates appropriate and professional non-verbal communication skills			
	**Total Hours**			

TUTOR SIGNATURE: .... **DATE:** ....
